# Gender-related psychosomatic peculiarities of patients with type 2 diabetes mellitus

**DOI:** 10.1192/j.eurpsy.2021.679

**Published:** 2021-08-13

**Authors:** N. Chernus, L. Kamynina, R. Gorenkov, A. Sivkov

**Affiliations:** 1 Department Of Outpatient Therapy, I.M. Sechenov First Moscow State Medical University: Moscow, Russia, Moscow, Russian Federation; 2 Endocrinology, Russian Medical Academy of Continuous Professional Education, Moscow, Russian Federation; 3 Scientific Substantiation Of Health Preservation Of The Population Of The Russian Federation, The N. A. Semashko National Research Institute of Public Health, Moscow, Russian Federation; 4 The Department Of Clinical Pharmacology And Internal Diseases Propaedeutics, the I.M. Sechenov First Moscow State Medical University, Moscow, Russian Federation

**Keywords:** gender, anxiety, depression, alexithymia

## Abstract

**Introduction:**

The problem of specificity of psychological adaptation mechanisms at the patients with Type 2 Diabetes Mellitus (T2DM) is extremely actual. The aim is to investigate gender psychological characteristics associated with T2DM

**Objectives:**

In the comparative study 62 patients (28 male, 34 female; mean age 56,8±2,3 and 55,4±2,7 yrs.) with T2DM (HbA1c 7,3±1,3%) and visceral obesity (Grade 2) were included.

**Methods:**

Research methods: the Depression Scale of Zung, the Spielberger trait scale anxiety, Toronto Alexithymia Scale and MMPI test

**Results:**

T2DM-female-patients in comparison with T2DM-male showed significantly higher personal anxiety scores (51,2+7,6 and 44,1+10,6 respectively; р<0,05), depression scores (44,2+7,6 and 36,7+8,4 respectively; р<0,05), while alexithymia scores were higher at T2DM-males (68,2+9,6 and 71,7+6,4 respectively; p<0,05). In MMPI test (after correction by K-scale) 46,8% of patients demonstrated profiles with elevated scale 1 score (above 70 Т-scores, but below 80 Т-scores) regardless of gender differences. However, the T-scores for T2DM-male patients were on the average by 1,07 higher than for T2DM-female (58,4 vs 54,4 respectively, р˃0,05), that indicated more higher concern related to own physical health condition. The female T2DM-patients significantly more often demonstrated profiles with scale 6 peak (exceeding 65 Т-scores): 79,4% vs. 21,4%, which indicated the more higher accentuation of personality traits (concealed hostility; protest; rigidity, desire to blame the others for one’s failure, et cetera)

**Conclusions:**

The patient’s gender has to be taken into consideration at development of clinical, diagnostic and prevention activities of patients with T2DM and visceral obesity.

